# Development of a Rapid, Antimicrobial Susceptibility Test for *E. coli* Based on Low-Cost, Screen-Printed Electrodes

**DOI:** 10.3390/bios10110153

**Published:** 2020-10-23

**Authors:** Stuart Hannah, Alexandra Dobrea, Perrine Lasserre, Ewen O. Blair, David Alcorn, Paul A. Hoskisson, Damion K. Corrigan

**Affiliations:** 1 Department of Biomedical Engineering, University of Strathclyde, 40 George Street, Glasgow G1 1QE, UK; alexandra.dobrea.2016@uni.strath.ac.uk (A.D.); perrine.lasserre@strath.ac.uk (P.L.); ewen.blair@strath.ac.uk (E.O.B.); damion.corrigan@strath.ac.uk (D.K.C.); 2 Division of Anaesthesia, Royal Alexandra Hospital, Corsebar Road, Paisley PA2 9PN, UK; David.Alcorn@ggc.scot.nhs.uk; 3 Strathclyde Institute of Pharmacy and Biomedical Sciences, University of Strathclyde, 161 Cathedral Street, Glasgow G4 0RE, UK; paul.hoskisson@strath.ac.uk

**Keywords:** antibiotic susceptibility testing (AST), *Escherichia coli* (*E. coli)*, electrochemistry, screen-printed electrodes (SPEs), streptomycin, growth-profiles, real-time monitoring

## Abstract

Antibiotic resistance has been cited by the World Health Organisation (WHO) as one of the greatest threats to public health. Mitigating the spread of antibiotic resistance requires a multipronged approach with possible interventions including faster diagnostic testing and enhanced antibiotic stewardship. This study employs a low-cost diagnostic sensor test to rapidly pinpoint the correct antibiotic for treatment of infection. The sensor comprises a screen-printed gold electrode, modified with an antibiotic-seeded hydrogel to monitor bacterial growth. Electrochemical growth profiles of the common microorganism, *Escherichia coli* (*E. coli*) (ATCC 25922) were measured in the presence and absence of the antibiotic streptomycin. Results show a clear distinction between the *E. coli* growth profiles depending on whether streptomycin is present, in a timeframe of ≈2.5 h (*p* < 0.05), significantly quicker than the current gold standard of culture-based antimicrobial susceptibility testing. These results demonstrate a clear pathway to a low cost, phenotypic and reproducible antibiotic susceptibility testing technology for the rapid detection of *E. coli* within clinically relevant concentration ranges for conditions such as urinary tract infections.

## 1. Introduction

Since their discovery in 1928, antibiotics treat bacterial infections and enable safe routine surgeries by minimising the risk of infection-related complications [1]. However, poor antibiotic stewardship spanning several decades across numerous industries such as agriculture [2] and cosmetics [3], has resulted in a vastly increasing prevalence of antibiotic resistant organisms.

The World Health Organisation (WHO) raised antibiotic, or more broadly, antimicrobial resistance (AMR) as an issue of upmost global importance [4,5], supporting Lord Jim O’Neill’s estimation of 10 million annual deaths from AMR by 2050, more than cancer and diabetes combined [6]. AMR risks returning medicine to a pre-antibiotic age, where routine surgeries become potentially life-threatening procedures.

Constant exposure to antibiotics selects for adaptive mutations that favour resistant strains [7]. Therefore, various methods to mitigate the spread of antibiotic resistance exist, including new drug and vaccination development, improved antibiotic stewardship and better diagnostic testing. Effective tackling of AMR will require a multipronged approach. Developing rapid and reliable point-of-care (POC) diagnostic tests will enable a quick identification not only of the type of infection (bacterial/nonbacterial), but also of the most effective (narrow spectrum) antimicrobial treatment required [8].

The current gold standard for antibiotic susceptibility testing (AST) involves culturing a sample to perform pathogen identification (ID) and then employing traditional microbiological techniques [9]. This process can often take at least 2 days (longer for slower growing organisms such as *Mycobacterium tuberculosis* (*M. tuberculosis*)), which can lead to an increase in resistance and even be potentially fatal in time-limited situations (e.g., sepsis). Automated systems exist based on disk diffusion and broth dilution methods where time-to-result is decreased to ≈6–12 h and throughput is significantly increased but these often require a dense bacterial suspension [10,11,12]. Current AST methods are not efficient enough for modern demands, adequate care of critically ill patients and are not compatible with rapid screening at the POC. There is therefore a real need for new POC diagnostic technologies which can rapidly assess the nature of an infection and identify the most suitable course of treatment to prescribe in timescales much quicker than current methods provide.

With the World Health Organization calling for a One Health approach [4] and subsequent private and public funding contributing to AMR research [13], a landscape of possibilities exists for AST innovation. On the contrary to high-throughput AST systems development, original proof-of-principle AST systems are flourishing [10]. Among many, Son and Stocker developed an artificial intelligence (AI)/machine learning (ML) concept based on single-cell motility and imaging to identify bacteria and assess their antibiotic susceptibility significantly speeding up AST [14,15]. Imaging coupled to a microfluidic chip has also proved to be very efficient for fast AST for urinary tract infections (UTIs) [16]. Nevertheless, the World Bank reframing AMR as a development challenge highlights that most recently proposed AST technologies do not progress towards further development, commercialisation and wider use because they require financial, laboratory or technological resources not affordable by all [13].

Electrochemical sensing systems offer many advantages looking towards AST diagnostics including low-cost, mass-manufacturable production, label-free detection and ease of integration with readout electronics [17] to provide useful sample-to-answer data for clinicians at the POC. Electrochemical impedance spectroscopy (EIS) is an important tool using a range of frequencies to gain information about both the resistive and capacitive (dielectric) properties of a system [18]. Investigation of impedance changes enables the assessment of bacterial growth over time [19,20]. Previously, EIS has been used to identify microorganisms including *Staphylococcus aureus* (*S. aureus*), *Escherichia coli* (*E. coli*) and *Pseudomonas aeruginosa* (*P. aeruginosa*) [20,21,22]. Discrimination between drug-susceptible *S. aureus* (MSSA) and the drug-resistant strain, MRSA, using low-cost, commercially available, screen-printed electrodes was shown in <45 min [23]. This work however was limited to organisms which displayed measurable growth changes before the gel-modified electrode dried out.

Screen-printed electrodes (SPEs) offer a number of advantages over more traditional electrode formats (e.g., three-electrode cells encompassing individual counter, reference and working electrodes). Advantages include low cost and ease of fabrication, simple cleaning processes, integration of the three-electrode cell onto an on-chip format, and they provide repeatable and reliable measurements with rapid time-to-result [24,25]. SPEs are particularly valuable for both prototyping and integration into rapid POC diagnostics since they can be mass-produced at relatively low cost compared to other types of macroelectrode or microelectrode [26]. In addition to bacterial detection, SPEs have previously been used for a wide range of applications including uric acid detection [27], glucose monitoring [28] and electrochemiluminescence (ECL) [29].

In this work, the concept of the gel-modified SPE [23] has been advanced by improving the measurement duration and assessing its potential for detecting a wider range of organisms. *Escherichia coli* is a Gram-negative bacterium commonly found in the mammalian gut and is considered part of the normal flora [30]. Although most *E. coli* are harmless, some can cause severe food poisoning [31] or facilitate resistance transmission through their capacity as a reservoir for resistance genes acquired by horizontal gene transfer [32]. *E. coli* is also a common cause of the bladder infection cystitis, which can lead to UTIs [33]. Like any microorganism, being able to identify it quickly and ascertain the best source of treatment is of upmost importance for patient health. To assess AST for *E. coli*, electrochemical growth profiles have been established using electrodes modified with gels with and without the antibiotic streptomycin present.

In addition to *E. coli* AST, the development of a test support structure is presented which enables the monitoring of bacterial growth over several hours without the limitation of the gel evaporating before significant microorganism growth has occurred (≈2 h for 50 µL). This will expand the utility of our system to capture a wider range of growth rates exhibited by pathogenic bacteria. For example, several mycobacteria feature doubling times on the scale of several hours to days [34], and the presented test support will enable AST to be performed on these types of organisms in addition to relatively fast-growing organisms such as *S. aureus,* and *E. coli* which is shown in this paper. The test support structure also provides the added advantage of levelling the baseline compared to the previous sensor variant without the test support [23], enabling changes due to bacteria growth/antibiotic action to be more readily distinguished from the growth curves.

## 2. Materials and Methods

### 2.1. Methodology

Commercially available gold (Au) screen-printed electrodes (SPEs) featuring on-chip silver reference and gold counter electrodes were used throughout the study (DropSens, Oviedo, Spain) (ref C223BT). Prior to use, SPEs were electrochemically cleaned by cyclic voltammetry (CV) between −0.3 and 1.5 V in 100 mM H_2_SO_4_ for approximately 10 scans (or until the CV was stable). After cleaning, electrodes were rinsed with deionised (DI) water and dried using argon.

Gel deposits were nominally produced in 100 mL batches and contained 1% agarose, 2.5 g Miller Lysogeny Broth (LB) Broth, 200 mM potassium chloride (KCl) +1 mM Fe${\left[\mathrm{CN}\right]}_{6}^{3-}$ +1 mM Fe${\left[\mathrm{CN}\right]}_{6}^{4-}$ (Ferri-Ferro Cyanide (FF-C) solution) in DI water. Some gels also contained streptomycin (~Minimum Inhibitory Concentration (MIC) 4 µg/mL). All chemicals were purchased from Sigma Aldrich (Dorset, UK). Gels were prepared at room temperature and autoclaved at 121 °C for 15 min for sterilisation and allow the components to mix. Gels harden upon cooling and were therefore stored in a water bath at 48 °C to maintain liquid form prior to deposition on the electrodes. Antibiotics were added immediately prior to deposition to avoid inactivation at elevated temperature.

*E. coli* (ATCC 25922) was streaked out onto plates containing LB media and agar (Sigma Aldrich) from a freshly prepared frozen glycerol stock. Upon growth on LB/Agar plates, single colonies were used to inoculate overnight cultures of LB (50 mL at 37 °C). Bacteria from the overnight cultures were pipetted directly onto the gel-modified electrodes (5 µL) at a concentration of ≈3.5 × $10^{9}$ CFU/mL, typical of an overnight bacterial culture, producing a starting *E. coli* count on the sensor of ≈1.75 × $10^{7}$ CFUs. Baseline measurements were performed in a similar manner, except the 5 µL overnight culture was substituted for 5 µL of LB medium with no bacteria as a negative control. Streak plates containing gel components contained LB but replaced agar with agarose to match the gel components.

Bacterial electrochemical growth profiles were measured for ≈5 h and each experiment involved three SPEs: a baseline measurement (gel only, i.e., no bacteria), gel + antibiotic with bacteria and gel (no antibiotic) with bacteria. Measurements were performed at 37 °C in the test support structure contained within an incubator (Genlab Ltd., Widnes, UK). EIS measurements were performed every 10 min using a measurement script and extracted parameters (e.g., *Z* at 100 kHz) were plotted as a function of time up to a maximum of ≈5 h growth post bacterial culture deposition. Each experiment was performed in triplicate.

### 2.2. Test Support Structure Design

The test support structure was developed principally to maintain gel integrity over prolonged time periods allowing for an extended AST measurement window that can capture the growth of microorganisms with longer doubling times (days/weeks) than those on the scale of minutes. The final test support design consisted of three main parts: a stainless steel base plate with slots for up to six SPEs (allows multiplexing of six SPEs concurrently), a hydrogel enclosure plate (transparent acrylic) and a top lid structure (transparent acrylic) to seal the samples and prevent gel drying. The acrylic parts were manufactured using a laser cutter (LPKF ProtoLaser). The base plate and the hydrogel enclosure plate were held together using a combination of countersunk M5 metal screws and rubber O-rings (d = 0.8 cm, 2 mm-thick), whereas the lid fitted onto the structure via magnets which were glued securely onto the lid. Figure S1 shows computer-aided design (CAD) drawing schemes of the test support components. The hydrogel enclosure mould is detailed in Figure S2. Finally, photographs of the final test support showing the assembly steps are shown in Figure S3. Before performing electrochemical growth measurements, the structure was tested by depositing 500 µL of water into the chambers and leaving it for several days to ensure an adequate seal was achieved, preventing evaporation.

### 2.3. Characterisation

**Figure 1:**
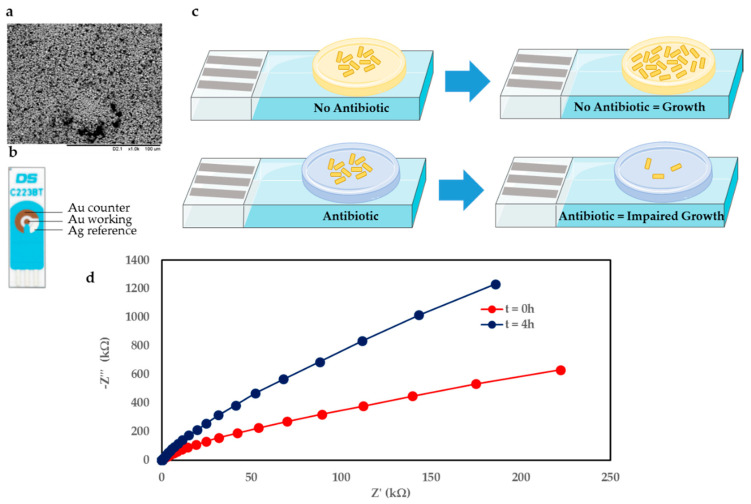
(**a**) SEM image of Au working electrode. (**b**) Schematic of Au DropSens SPE featuring counter, reference and working electrodes. (**c**) Overview of sensor technology showing the effect of *E. coli* on a gel-modified SPE containing no antibiotic (above) and antibiotic (below) over time. (**d**) Electrochemical Impedance Spectroscopy (EIS) traces comparing scenario at t = 0 h (initial condition) with t = 4 h (after 4 h of bacteria growth on gel containing no antibiotic).

Electrochemical measurements on the SPEs were performed using a three-electrode cell as shown in Figure 1b. Measurements were performed using a potentiostat (PalmSens PS4, PalmSens, Houten, Netherlands) with associated data analysis software (PSTrace).

Scanning electron microscope (SEM) (TM-1000, Hitachi, Tokyo, Japan) images of the Au working electrode SPE surface were performed by scanning a 150 × 150 µm-area at ×1.0 k magnification (Figure 1a).

Bacterial growth profiles were characterised by electrochemical impedance spectroscopy (EIS) across a frequency range between 100 kHz and 0.1 Hz at open-circuit potential (OCP) (≈0.3 V). Whilst it is common to fit EIS data to an equivalent circuit such as the Randles’ Equivalent Circuit, we have found that our set-up can discern bacterial growth trends directly from ‘raw’ impedance values at a particular frequency (not requiring a circuit model) ultimately decreasing the instrumentation complexity which facilitates ready deployment at the POC. Various parameters were examined and both *Z* and the phase angle at 100 kHz were chosen as the most representative indicators of bacterial growth. The electrochemical parameters were normalised by dividing each data set by their corresponding value at time t = 0 as described by Connolly and Shedden (2010 Patent) [35]. Independent two-tailed *t*-tests were then performed to compare the parameters recorded in the presence or absence of antibiotics at each time point (*n* = 3).

## 3. Results and Discussion

### 3.1. Sensor Overview

In contrast to genotypic antibiotic resistance tests (ART) calling for prior knowledge of resistance determinants, phenotypic AST suggests antibiotics that would be effective against the micro-organisms tested [36,37]. The gel-modified electrodes enable different types of bacterial infections to be detected and the effect of antibiotics in their presence. In this case, the common infection *E. coli* was chosen as the bacteria of interest. Figure 1c shows an overview of the sensor principle, whereby the gel-modified electrode effectively represents a miniaturised agar plate on an electrode sensor. The case featuring no antibiotic in the gel vs. gel seeded with antibiotic is shown in Figure 1c above and below, respectively. Upon deposition of *E. coli* onto the gel, for the scenario where no antibiotic is present, the bacteria are able to grow unhindered on the electrode over time. However, when the gel is seeded with antibiotic at a concentration greater than the minimum inhibitory concentration (MIC), the antibiotic causes bacterial growth to be hindered, which is reflected in the electrochemical measurements performed in real time.

The measurement setup consisted of a gel-modified SPE connected to a potentiostat controlled by associated measurement software. This setup can be scaled up to simultaneously monitor several (≤8) electrodes in real time using a multiplexer format.

A commercially available electrode was used in this study since it is low cost (<£2) and can easily be integrated with the existing measurement setup. The electrode combines Au counter (CE) and working electrodes (WE), with a Ag reference electrode (RE). The working electrode is 1.6 mm in diameter. Figure 1b shows the electrode used, and an SEM image of the Au WE surface in Figure 1a. The SEM image shows that the Au surface is highly irregular and features deep voids and nonhomogenous particle sizes.

Figure 1d shows exemplar electrochemical impedance spectroscopy (EIS) Nyquist plots for the gel featuring no antibiotic with *E. coli*. The traces compare the initial condition immediately after bacteria deposition (t = 0 h) and after 4 h of bacteria growth on the gel-modified electrode (t = 4 h).

### 3.2. Standardisation Experiments

The major factor responsible for accelerating hydrogel drying in previous experimental work was the high temperature incubating conditions employed to promote bacterial growth (37 °C). To minimise the amount of hydrogel moisture loss over the course of an experimental run, two possible strategies were explored:

1. Increase the air water vapour content and thus balance the evaporation rate by gel environmental water absorption (hydrogels are highly hygroscopic structures, prone to ‘swelling’ in humid environments).

2. Enclose the gel samples within a smaller volume to induce the system to quickly reach saturation (condensation and evaporation rates become equal) and therefore expose the gel to a consistent moisture level and effectively cause zero net evaporation.

**Figure 2:**
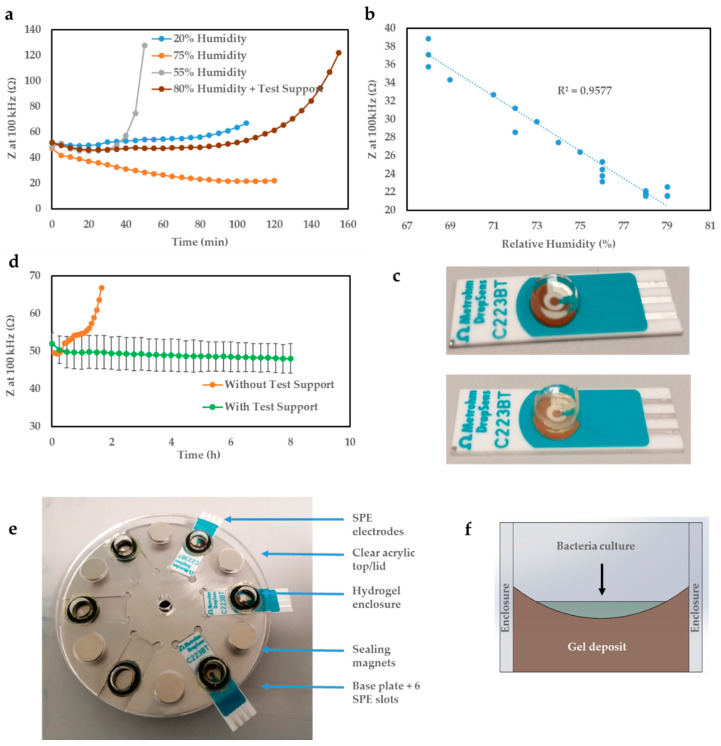
(**a**) Summary of humidity control experiments showcasing the effect on environmental humidity level on gel electrochemical impedance at 100 kHz, the sharp increase in impedance evidencing the hydrogel drying out as time elapses except at 75% (orange curve). (**b**) The 75% humidity measurement indicating a strong linear dependence (R^2^ = 0.95) between hydrogel impedance and environmental humidity level (after allowing 10 min for humidity stabilisation). (**c**) Photograph of Au DropSens electrodes modified with gel-deposit before measurement (above), and after 8 h baseline measurement with the test support (below). (**d**) Electrochemical baseline data with gel-only comparing *Z* at 100 kHz over time with and without test support (*n* = 3 SPEs). (**e**) Test support structure created to maintain gel integrity with SPEs and (**f**) schematic of test support hydrogel enclosure showing gel deposit and bacteria culture.

Implementing the first strategy involved placing a humidifier inside the incubator which would automatically adjust the humidity level based on the desired humidity setting. A baseline (no bacteria) electrochemical measurement was performed simultaneously to investigate the effect of humidity on the resulting electrochemical data. These experiments were only exploratory and therefore were not replicated. The resulting impedance traces obtained following the humidity experiments were reproduced in Figure 2a. Initial experiments revealed that during normal operation, the humidity level inside the incubator was maintained constant at 20% relative humidity (RH). Prolonged exposure to this humidity level would be problematic not only because of gel drying which would destabilise the impedance traces leading to a sharp increase in magnitude, but also for bacterial and fungal growth in general which preferentially takes place in humid conditions.

Increasing the RH level beyond this would not only slow down the evaporation rate by increasing the air humidity ratio, but also promote water attachment to the polymeric backbone of the hydrogel. However, it was found that electrochemical parameters such as the impedance modulus, which historically was shown to be a useful means of quantifying bacterial growth and metabolic activities, was very sensitive to humidity variations (Figure 2b). At 55% RH (±12% SD), the impedance followed the humidity trace for the entirety of the testing window with only a small time delay in-between (not reproduced). This time delay was likely related to the time required for the RH level to stabilise inside the incubator following humidity adjustment by the humidifying element. When working with a 75% RH level, a measurement where the humidity was much more stable inside the incubator (±4% SD), a strong linear dependence between the gel electrochemical impedance and environmental humidity was noticed (Figure 2b). Interestingly, the 55% RH measurement appear to be significantly less stable over time compared to 20% and 75%. This is likely related to the setup of the humidifying element, and its ability to maintain a stable humidity at that level. If the measurement were to be repeated, adequate time would be left prior to measuring to ensure the humidity level had stabilised adequately prior to recording data.

Additionally, if left free-standing inside the incubator in a highly humid environment, the gel was found to swell as indicated by the decreasing impedance modulus value at 100 kHz (75% measurement) until it would eventually collapse introducing the additional variable of significant morphological change.

Even when enclosed within an unsealed support frame to maintain its structural integrity, the hydrogels would still evaporate in highly humid conditions (80% ± 7% RH) likely as a result of the evaporation rate surpassing that of water absorption when the effective air exposed area was reduced (rather than a dome, the support gave rise to a ‘well’ type structure due to wall attachment). These results suggested that, in order to maintain a consistent baseline for bacterial measurements, the humidity level the hydrogel is exposed to during incubation must remain relatively constant throughout the entire duration of the testing period, ideally in the range 90–100% RH. A cheaper and likely more effective alternative to optimal, sensitive humidity control would be completely enclosing the hydrogel within a sealed test support to essentially create an atmosphere of zero net evaporation.

Figure 2e displays the test support structure developed to be able to monitor bacterial growth curves for a longer period of time (several hours) compared to ≈2 h previously possible without the test support, due to the gel drying. The stainless-steel base and acrylic enclosure and lid are inexpensive and permit aseptic cleaning with 70% ethanol. In addition, acrylic is convenient to manufacture and shows electrochemical inertia. The gel enclosure developed is highlighted in Figure 2f and shows the way the gel forms in the well. This specific shape results from the hydrophilic interaction of the hydrogel with acrylic through wall attachment. Compared to the previous dome-shaped gel formation [23], it ensures the full bacterial deposit is contained and tested. An example electrode with the gel in place before baseline data was recorded is displayed in Figure 2c (top) and upon test support removal after an 8 h baseline measurement (below) for comparison. It is clear that the gel maintains its integrity and keeps the distinctive ‘well’ shape across the entire 8 h measurement window.

Using the test support, whilst the gel still evaporates, assuming a perfectly sealed enclosure, condensation balances out that evaporation resulting in zero net evaporation. This in turn enables the establishment of a very flat baseline curve as shown in Figure 2d.

With the support in place, the gel-modified sensor displays an impedance (modulus) (*Z*) at 100 kHz of ≈50 Ω, which remains stable for the entire 8 h under observation. On the contrary, the same measurement without the test support shown in orange, starts around 50 Ω, but then steadily climbs to almost 70 Ω before fully evaporating within 2 h. Therefore, the test support brings a better consistency in the deposition of the hydrogel and bacterial sample onto the electrode measuring area, enhancing repeatability of the presented AST technology.

As a result of the promising data shown using the test support, principally its ability to maintain a steady baseline over a significantly longer period of time, subsequent growth profiles with *E. coli* were performed using the support. The test support provides the ability to monitor organisms with longer doubling times enabling the development of a truly generic AST sensor for any type of bacterial/fungal sample presented.

### 3.3. Bacterial Growth Profiles

**Figure 3:**
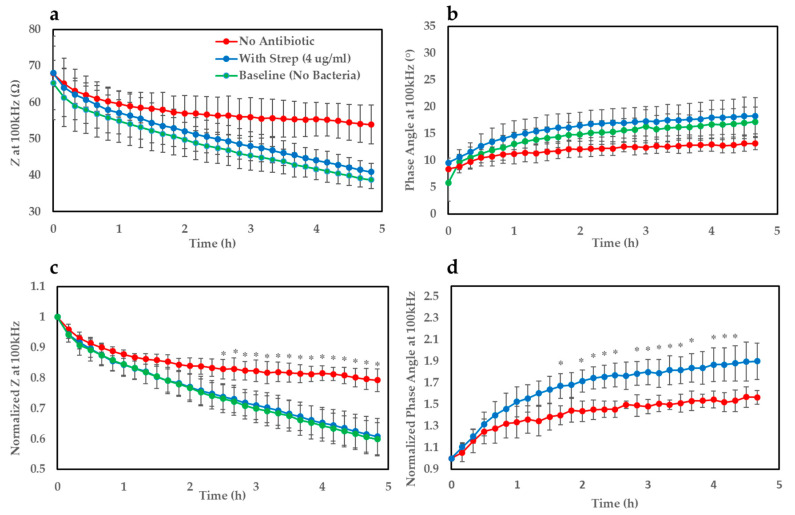
(**a**) Bacterial growth curves (*n* = 3 SPEs) of *Z* at 100 kHz of *Escherichia coli* (ATCC 25922) on gels seeded with and without streptomycin (4 µg/mL) and baseline curve (no bacteria). (**b**) Bacterial growth traces of phase angle at 100 kHz and baseline measurement (no bacteria). (**c**) Growth curves of normalised *Z* at 100 kHz. Growth curves for gels with and without streptomycin and baseline. (**d**) Normalised phase angle at 100 kHz for the gels with and without streptomycin.

Upon establishment of the test support, the next step involved validation of the structure with *E. coli* which involved depositing a small volume (5 µL) of an overnight culture of *E. coli* onto a gel-modified electrode and monitoring electrochemical growth profiles over time. EIS was performed every 10 min and various parameters including *Z* at different frequencies were extracted and plotted over time. Similarly to the case with *S. aureus*/MRSA [23], *Z* at 100 kHz appeared to be the most sensitive parameter to monitor changes in bacterial growth. Figure 3a shows growth curves (*Z* at 100 kHz) of *E. coli* on the gel-modified electrodes for ≈5 h. The impedance traces were normalised with respect to their corresponding value at time t = 0 which allowed a clearer distinction to be observed (Figure 3c). It became apparent from the profiles that *E. coli* deposited onto the gel seeded with streptomycin (~MIC–4 µg/mL) shows a very similar growth trend to the baseline curve where no bacteria was added (5 µL of LB only to mimic overnight culture). On the other hand, for the case where no antibiotic was present in the gel, the gradient of the growth curve appears flatter over time indicating a steady increase in impedance compared to the baseline and antibiotic-infused gel, and begins to show statistical significance (*p* < 0.05) after ≈2.5 h. A similar effect could be noticed clearly for the phase angle parameter (Figure 3b) after normalising the bacterial traces with respect to the measurement taken at time t = 0 (Figure 3d). This parameter produced a faster time-to-result of around 1 h and 40 min, but suffered from higher variation, possibly due to electrical noise. This resulted in very large interexperimental standard deviations for the baseline parameter, hence why the baseline curve has been omitted in this case. This variation is likely the explanation as to why not all time points after the detection threshold were significantly different between the bacterial traces with and without antibiotic. Regardless, this parameter still proved to be a valuable indicator of bacterial growth and metabolic activity.

**Figure 4:**
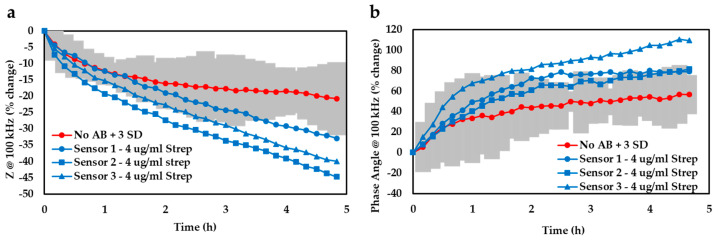
The % change at 100 kHz of (**a**) impedance and (**b**) phase angle for gels with and without streptomycin.

In addition to the data normalisation approach, we also determined the limit of detection afforded by our device by plotting the 99% confidence zone surrounding the *E. coli* trace and noted the experimental timeframes required for the antibiotic-infused bacterial traces (*n* = 3) to diverge from that zone (Figure 4). By averaging these temporal values, it was found that our detection system could successfully discern bacterial growth after only ≈2.5 h based on the impedance readout at 100 kHz (a) or ≈2.1 h if using the phase angle at 100 kHz (b). Both analysis techniques considered in this study thus yielded comparable time-to-results and either could potentially represent a valuable threshold detection mechanism that a future device might be based on.

This time-to-result is significantly quicker than the current gold standard AST of at least 1–2 days, and could be a very useful tool for rapid, low-cost AST testing at the point of care (POC). While it is envisioned that going forth, we may further decrease the experimental timescales by implementing chemometric techniques such as principal components analysis (PCA) or machine learning algorithms, in this case we opted for a more simplistic form of data analysis (i.e., normalisation and computing the 3 SD zone) to decrease the overall system complexity and make it more amenable for use at the POC. Such a POC device could be used by clinicians to rapidly choose the best antibiotic to treat a particular infection, in timescales much quicker than current methods enable. A test like this one would vastly improve patient health, as well as help avoid the unnecessary prescribing of (typically broad spectrum) antibiotics whilst improving stewardship of our most treasured antimicrobial stocks.

EIS was chosen over alternative electrochemical techniques such as voltammetric or amperometric techniques for a number of reasons including its sensitivity, and for the large amount of data that can be produced in a single measurement across a wide frequency range in a relatively short period of time. When looking towards a final POC device, being able to simplify the measurement electronics to a smaller frequency range, or indeed performing a DC measurement could be invaluable for reducing device complexity and overall cost. However, during the development stage, collecting vast amounts of EIS data is useful in order to establish the optimum parameter/frequency indicative of bacteria growth.

The phenotypic nature of this technology means it is highly versatile and can be used for a wide range of pathogenic organisms (Gram-positive or -negative bacteria) including ‘slower’ growing organisms such as *M. tuberculosis* to provide a rapid time-to-result for AST. Furthermore, our method has high-sensitivity enabling detection of *E. coli* at clinically relevant concentrations, given the UTI bacterial threshold is ≥$10^{5}$ CFU/mL, and typically anywhere up to $10^{8}$ CFU/mL, and *E. coli* is the most common uropathogen [38,39]. Further work will look to improve test sensitivity down to at least 10^5^ CFU/mL, but the technology is currently well within the acceptable range for a positive UTI diagnosis. This technology may also find utility in testing clinically relevant fungi such as *Candida albicans* and *Cryptococcus* species.

Fast time-to-result with a simple measurement format at the point-of-care could inform the therapeutic decision independently from resource availability and enhance overall antibiotic stewardship. Moreover, it would allow the monitoring of treatment efficacy for a more personalised, therapeutic approach to improve patient outcomes, maintain antimicrobial efficacy, reduce antimicrobial resistance associated costs and mitigate the spread of AMR.

## 4. Conclusions

A rapid, low cost, electrochemical sensor was developed to monitor bacterial growth over time. The sensor consists of gel-modified screen-printed electrode sensors capable of measuring antibiotic susceptibility profiles of the common infection *E. coli*. Gels with and without 4 µg/mL streptomycin were deposited onto electrodes, and *E. coli* growth was monitored over time. As expected, no growth was observed in the presence of antibiotic, however, where no antibiotic was present, *E. coli* was able to grow, and a clear difference between the two growth profiles was observed in ≈2.5 h, a significant reduction in the current gold standard techniques for AST of ≈1–2 days. In addition, a test support structure to house the electrode sensors enables growth profiles to be recorded over several hours, a vast improvement on the stand-alone electrodes which suffer from gel drying effects after ≈2 h. This was a much necessary modification to be able to monitor slower growing microorganisms such as *M. tuberculosis* at the POC. These developments represent a clear step forward towards widespread, low cost and routine antibiotic susceptibility testing which will be critical in the future, where antibiotic prescriptions might not be possible without a confirmatory test due to e.g., government legislation.

## Supplementary Materials

The following are available online at www.mdpi.com/xxx/s1, Figure S1: CAD drawing schemes of test support components (a) and assembly (b); Figure S2: Hydrogel mould concept. (a) Isometric view of CAD drawing and (b) Section view of individual hydrogel enclosure unit; Figure S3: Photographs of the finalised test support showing assembly steps (a) Separate parts with lid on the left, hydrogel mould on the right and base plate in the middle where (b) SPEs are placed. (c) The base plate and hydrogel enclosure are screwed together and (d) The lid is placed on top of the enclosure.
