# Supplementary material for: Development of a Rapid, Antimicrobial Susceptibility Test for E. coli Based on Low-Cost, Screen-Printed Electrodes
Source: Biosensors (Basel). 2020 Oct 23;10(11):153. doi: 10.3390/bios10110153 (PMC7690799; doi:10.3390/bios10110153)
Supplement: Supplementary file 1 [file biosensors-10-00153-s001.pdf]

biosensors

MDPI

Supplementary Materials

Article

Development of a rapid, antimicrobial susceptibility test for *E. coli* based on low-cost, screen-printed electrodes

Stuart Hannah [1,†,*], Alexandra Dobrea [1,†], Perrine Lasserre [1], Ewen O. Blair [1], David Alcorn [2], Paul A. Hoskisson [3] and Damion K. Corrigan [1]

[1] Department of Biomedical Engineering, University of Strathclyde, 40 George Street, Glasgow, G1 1QE, UK.; stuart.hannah@strath.ac.uk, alexandra.dobrea.2016@uni.strath.ac.uk, perrine.lasserre@strath.ac.uk, ewen.blair@strath.ac.uk, damion.corrigan@strath.ac.uk.

[2] Division of Anaesthesia, Royal Alexandra Hospital, Corsebar Road, Paisley, PA2 9PN, UK.; David.Alcorn@ggc.scot.nhs.uk

[3] Strathclyde Institute of Pharmacy and Biomedical Sciences, University of Strathclyde, 161 Cathedral Street, Glasgow, G4 0RE, UK; paul.hoskisson@strath.ac.uk.

* Author to whom correspondence should be addressed.

†These authors contributed equally to this work.

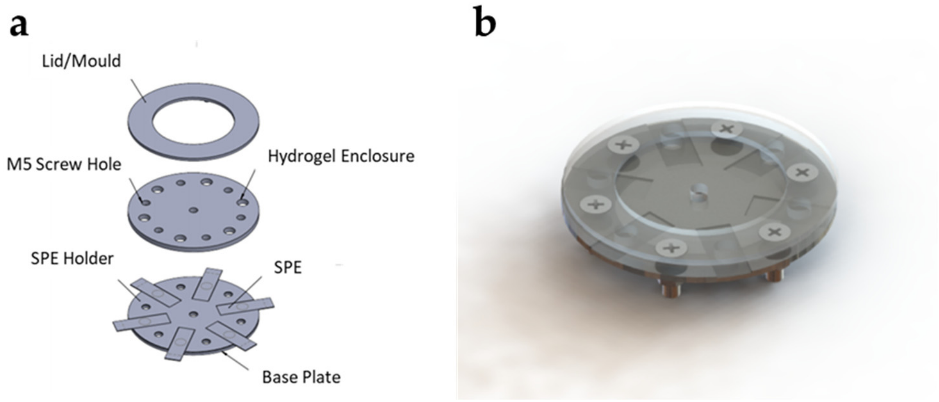

Figure S1. CAD drawing schemes of test support components (a) and assembly (b).

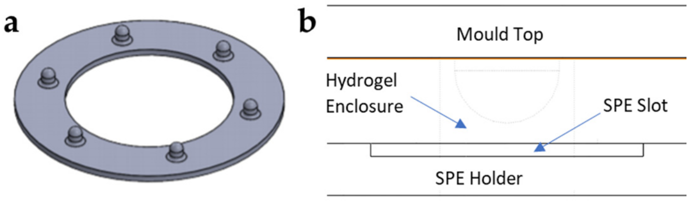

Figure S2. Hydrogel mould concept. (a) Isometric view of CAD drawing and (b) Section view of individual hydrogel enclosure unit.

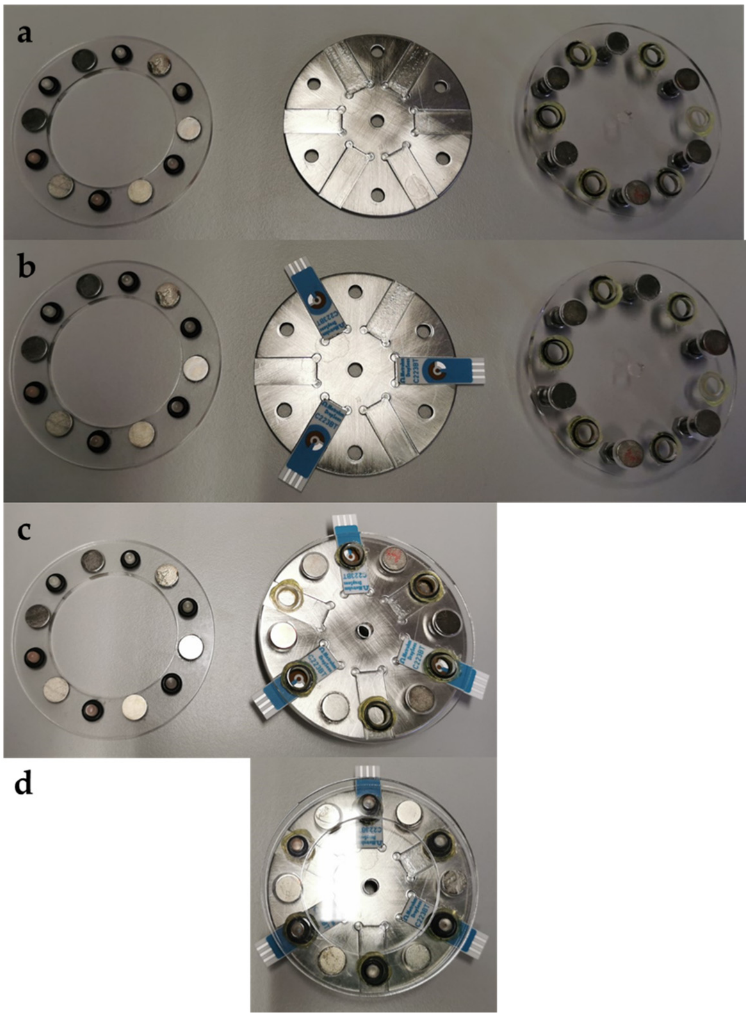

Figure S3. Photographs of the finalised test support showing assembly steps (a) Separate parts with lid on the left, hydrogel mould on the right and base plate in the middle where (b) SPEs are placed. (c) The base plate and hydrogel enclosure are screwed together and (d) The lid is placed on top of the enclosure.
